# Evaluation of the Nystagmus Information Pack

**DOI:** 10.22599/bioj.269

**Published:** 2022-09-15

**Authors:** Anne Bjerre, Helen Griffiths, Martha Foulds, Gemma Arblaster

**Affiliations:** 1 University of Sheffield, GB; 2 Sheffield Children’s NHS Foundation Trust, GB

**Keywords:** nystagmus, patient information, questionnaire

## Abstract

**Introduction:**

In response to the need for easily accessible, high-quality information about nystagmus, the Nystagmus Information Pack was created and made freely available online in 2017. This study was undertaken to evaluate the content and accessibility of the Nystagmus Information Pack.

**Methods:**

Clinicians, eye clinic liaison officers (ECLOs), teachers, patients, families, and any person with an interest in nystagmus were invited to complete an online questionnaire about the content and accessibility of the Nystagmus Information Pack.

**Results:**

One hundred and sixty respondents completed the questionnaire. Respondents who had previously accessed the Nystagmus Information Pack (n = 49, 30.6%) reported the content was appropriate (86%), of sufficient detail (94%), and easy to understand (88%). Minor suggestions were made to improve the content. Respondents who had not accessed the Nystagmus Information Pack (n = 111, 69.4%) reported not being aware of the resource (90%) but had already accessed nystagmus information from a wide range of sources. Poor vision was a barrier to accessing the resource for a small number of respondents (4.5%).

**Conclusion:**

Some improvements to the content and accessibility of the Nystagmus Information Pack should be considered, in particular the format options in which it is available, to enable access in preferred formats and with poor vision. The availability of the Nystagmus Information Pack should be promoted and shared more widely, as the majority of respondents were unaware of the resource despite having an association with or interest in nystagmus.

## Introduction

Nystagmus is an involuntary movement of the eyes, which can be infantile or acquired. Typically, nystagmus leads to reduced vision (Abadi & Bjerre 2002) and other negative experiences that impact quality of life (McLean, Windridge & Gottlob 2012). Bjerre et al. (2018) highlighted the need for more standardised and accessible information about nystagmus to be available to patients, families and clinicians. The Nystagmus Information Pack (2017) was developed by a team at the University of Sheffield, with input from service users (people with nystagmus and family members of people with nystagmus) and clinicians (see Part 1 of the Nystagmus Information Pack for a full list of contributors). In 2017, the Nystagmus Information Pack was made freely available online to specifically address the lack of high-quality standardised information about nystagmus. It was highly commended in the British Medical Association (BMA) Patient Information Awards in 2018. At this time, additional information about nystagmus was also made available by charities, including the Nystagmus Network (2018), the Royal National Institute of Blind People (RNIB) (2017), and Infantile Nystagmus (IN) – Vision (2021). Despite these improved sources of information about nystagmus, Gummer et al. (2020) found regional variation in access to information and support from educational vision support services and other sources of visual impairment support, such as from charities and support groups, still existed around the UK.

In 2019, Harris et al. highlighted the variation in investigation, diagnosis, and management of nystagmus in the UK. A nystagmus ‘care pathway’ was proposed in an attempt to ensure all patients had access to a minimum standard of care in line with current evidence whilst also avoiding unnecessary clinical visits or investigations (Harris, Owen & Sanders 2019). In 2020, a review of the management of nystagmus in children was published (Self et al. 2020) in a further attempt to standardise the investigation and management of nystagmus in children in the UK. The importance of providing patients and their families with high-quality information about nystagmus was emphasised by both Harris, Owen, and Sanders (2019) and Self et al. (2020).

The aim of this study was to evaluate the Nystagmus Information Pack (2017) using an online questionnaire, with particular focus on both the content of the information and its accessibility.

## Methods

Ethical approval for an online questionnaire evaluating the content and accessibility of the Nystagmus Information Pack was granted by the University of Sheffield (reference 034410). The questionnaire was created using Google Forms and distributed to a range of organisations and groups that were expected to have contact with people with nystagmus. These included clinicians (orthoptists, optometrists, or ophthalmologists), eye clinic liaison officers (ECLOs), teachers, charitable organisations, and support groups. Patients, families, and anyone with an interest in nystagmus were invited to complete the questionnaire. Social media accounts relating to the Division of Ophthalmology and Orthoptics, University of Sheffield, were also used to invite volunteers to complete the questionnaire.

Prior to completion of the questionnaire, respondents were asked to read information explaining the purpose of the questionnaire and provide informed consent for their responses to be used anonymously to evaluate the Nystagmus Information Pack and publish the results. The questions within the questionnaire are shown in Appendix 1. Respondents were encouraged to select all responses that were relevant. Free text boxes were used to encourage respondents to expand on or to provide additional detail to their answers, where they felt it was appropriate. The questionnaire was available for completion for one month, beginning 26 June 2020. Reminder information was sent one week before the deadline to maximise the response rate. Questionnaire responses were collated in a spreadsheet and analysed quantitatively. Free text qualitative responses were reviewed. Whilst insufficient detail was available for in-depth qualitative analysis, respondent information was used to support and enhance the interpretation of the quantitative data where possible.

**Figure 1:**
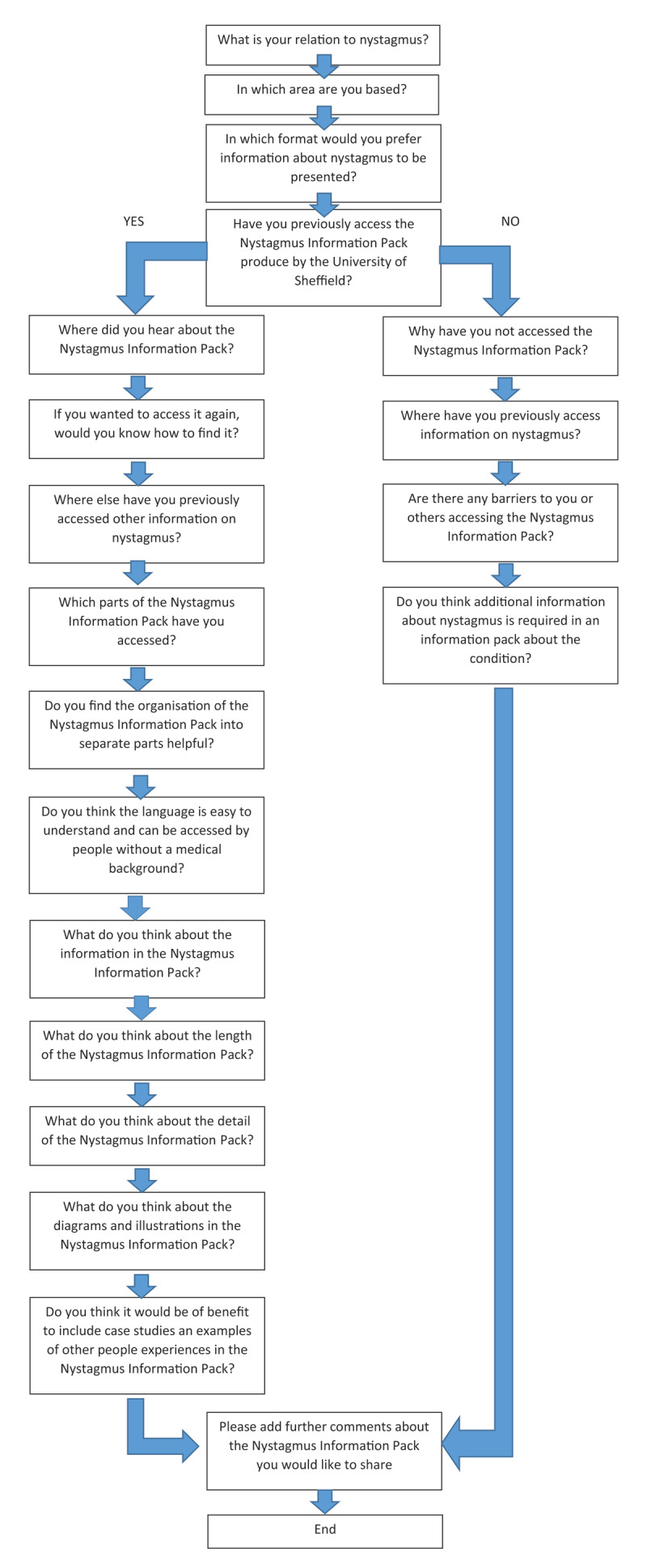
A flow chart to show the respondents’ route through the questionnaire.

The questionnaire (Appendix 1) was designed so that all respondents completed the first three questions. Based on whether respondents had accessed the Nystagmus Information Pack previously, or not, they then completed questions either about the accessibility and content of the Nystagmus Information Pack or about reasons for not accessing it and general questions about nystagmus information (Figure 1).

## Results

One hundred and sixty responses to the questionnaire were received. The majority (39%) of respondents were orthoptists, optometrists, or ophthalmologists (Table 1). There was also a high number of responses from people who had nystagmus (33%) or were family members of someone with nystagmus (18%). A small number of respondents selected more than one option; the majority of those were from people with nystagmus or family members of people with nystagmus who also worked in a vision-related field.

**Table 1:** Displays the responses to the question, What is your relationship to nystagmus?


QUESTION 2: WHAT IS YOUR RELATIONSHIP TO NYSTAGMUS?	NUMBER OF RESPONSES	PERCENTAGE (%) OF TOTAL RESPONSES

I have nystagmus	57	33%

I am a family member of someone with nystagmus	31	18%

I teach people with nystagmus	5	3%

I am an orthoptist, optometrist, or ophthalmologist	66	39%

I am an eye clinic liaison officer (ECLO)	6	4%

I am another health professional	2	1%

I work for a sight loss/disability organisation	3	2%

Student studying orthoptics	1	1%

**Total number of responses**	**171**	**100%**

There were 160 questionnaire respondents and 171 responses to question 2, as participants could select more than one option.		


The majority (72.6%) of respondents were from England, with every region represented. Smaller numbers of responses were received from Scotland (6.4%), Wales (5.7%), and Northern Ireland (1.3%). The remaining 14% of respondents were from outside the UK. The location of the respondents are shown in Table 2.

**Table 2:** Displays the responses to the question, In which area are you based?


QUESTION 3: IN WHICH AREA ARE YOU BASED?	NUMBER	PERCENTAGE %

England	114	72.6%

Scotland	10	6.4%

Wales	9	5.7%

Northern Ireland	2	1.3%

Republic of Ireland	3	1.9%

Germany	1	0.6%

Turkey	1	0.6%

USA	14	8.9%

Canada	1	0.6%

Pakistan	1	0.6%

New Zealand	1	0.6%

**Total**	**157**	**100.0%**

157 responses out of a total of 160 questionnaire respondents. No respondents selected multiple locations		


The majority of respondents (69%) preferred information about nystagmus to be published on a website. PDF (55%), video (52%), and printed information (48%) formats were also favoured (Table 3). The 66 orthoptists, optometrists, and ophthalmologists that completed the questionnaire strongly favoured websites (80%) followed by PDF (65%). Of those with nystagmus (*n* = 57), website information was preferred (61%) and then video (58%) and PDF (48%). People with nystagmus preferred a broader range of formats to clinicians. Websites were preferred due to ease of access and the most recent information being available. Clinicians and sight loss organisations reported they directed people to websites for easy and direct access to information that was updated regularly. PDF and print were preferred by clinicians and teachers due to ease of access and being able to physically hand out information. Individuals with nystagmus and their families reported printed information was also useful to give to others (e.g., school). They also liked being able to go over printed information at their own pace and the option to adjust PDF settings to enlarge the font size. Videos were reported as useful for showing to children and those who struggled to read and for sharing information with others (e.g., wider family). Audio information was reported as useful for those who had difficulty reading print and had a preference for listening to information. Many participants described having a range of formats in which they could receive information as useful.

**Table 3:** Displays the formats in which participants would prefer information about nystagmus to be presented.


QUESTION 5: IN WHICH FORMAT WOULD YOU PREFER INFORMATION ABOUT NYSTAGMUS TO BE PRESENTED?	ALL RESPONDENTS (*N* = 159)	ORTHOPTISTS, OPTOMETRISTS OR OPHTHALMOLOGISTS (*N* = 66)	PEOPLE WITH NYSTAGMUS (*N* = 57)

Video	82 (52%)	28 (42%)	33 (58%)

Audio	49 (31%)	19 (29%)	20 (35%)

Website	110 (69%)	53 (80%)	35 (61%)

PDF	87 (55%)	43 (65%)	28 (49%)

Print	76 (48%)	38 (58%)	20 (35%)

Don’t know	5 (3%)	1 (2%)	3 (5%)

Any	1 (0.6%)	0 (0%)	0 (0%)

**Total responses**	**410**	**182**	**139**


Out of 160 respondents, 159 completed question 5. Participants could select multiple options. A total of 410 responses were recorded.

Participants were asked if they had previously accessed the Nystagmus Information Pack (question 7). One hundred and eleven (69%) had not previously accessed the Nystagmus Information Pack, and 49 (31%) had accessed it. Responses from those who had previously accessed the Nystagmus Information Pack are presented, followed by those who had not accessed the Nystagmus Information Pack (Figure 1).

### Previously accessed the Nystagmus Information Pack (*n* = 49)

Those who had previously accessed the Nystagmus Information Pack (*n* = 49) proceeded to answer questions about the content (Table 4). The most commonly accessed part of the Nystagmus Information Pack was Part 5: Treatment options and long-term outcomes (98%), and the least commonly accessed part was Part 4: What to expect at eye clinic appointments (82%). Most respondents (92%) felt the organisation of the Nystagmus Information Pack into separate parts was helpful, with a small number responding that this was not helpful (6%) or they were unsure (2%).

**Table 4:** Parts of the Nystagmus Information Pack that had been accessed.


QUESTION 17: THE NYSTAGMUS INFORMATION PACK IS SPLIT INTO SEVEN PARTS. WHICH PARTS HAVE YOU ACCESSED?	TOTAL RESPONDENTS (*N* = 49)	PERCENTAGE (%)

Part 1: An introduction to nystagmus	45	92

Part 2: Infantile nystagmus	45	92

Part 3: Acquired nystagmus	44	90

Part 4: What to expect at eye clinic appointments	40	82

Part 5: Treatment options and long-term outcomes	48	98

Part 6: Useful contacts and information	45	92

Part 7: Information about nystagmus for families	47	96

**Total responses**	**314**	


All 49 respondents completed question 17. Participants could select multiple options. A total of 314 responses were recorded.

Most respondents (88%) reported the language used in the Nystagmus Information Pack was easy to understand and could be accessed by non-medical people (question 20). Most respondents reported the Nystagmus Information Pack was an appropriate length (86%; question 24) and level of detail (94%; question 26). A small proportion reported it was too long (14%) or too detailed (6%). Eighty-one percent reported the content of the Nystagmus Information Pack was appropriate, and 67% reported all the information in the Nystagmus Information Pack was needed. Most comments about the detail and content of the Nystagmus Information Pack were positive, as the separation into different sections allowed users to select the parts appropriate to them and to take away more detailed information about nystagmus. A small percentage reported that the Nystagmus Information Pack contained information that was not needed (6%), misleading (4%), or missing (2%; question 22). Comments were made about recent changes to UK requirements relating to driving and nystagmus and ensuring country-specific information was clearer for international users of the Nystagmus Information Pack. One clinician commented that pharmaceutical treatment, botulinum toxin (BT), or surgery for nystagmus was only offered in their area in severe and symptomatic cases, and there was concern the treatment section of the Nystagmus Information Pack could be interpreted as these treatments being widely available in all areas.

The diagrams and illustrations within the Nystagmus Information Pack were reported to be helpful (58%), appropriate (31%), or unhelpful (10%; question 28). Comments about the drawings included their not being very clear and colour or photographs being preferred over line drawings. Seventy-four percent reported that case studies and examples of other people’s experiences of nystagmus would be beneficial to include in the Nystagmus Information Pack (question 30). Comments related to the inclusion of case studies and examples highlighted the difficulty of providing real-life information about living with the condition when individual experiences with nystagmus are often variable. Some commented that including links to other resources and sources of information containing case studies and nystagmus support groups was more useful than additionally including individual examples in the Nystagmus Information Pack.

### Had not previously accessed the Nystagmus Information Pack (*n* = 111)

The main reason reported for not accessing the Nystagmus Information Pack was that ‘they had not heard of it’ (90%). Five respondents had heard of the Nystagmus Information Pack but were unable to find it or thought it was an ‘in-house’ resource. Six reported they did not need to access it (question 8). In those who had not accessed the Nystagmus Information Pack (*n* = 111), the most common sources of information about nystagmus that had been accessed were the charity Nystagmus Network (62%) and online searches (59%), followed by the hospital eye clinic (44%), social media (25%), and other sight loss charities (18%; question 9; Table 5).

**Table 5:** Where respondents had previously accessed information on nystagmus.


QUESTION 9: WHERE HAVE YOU PREVIOUSLY ACCESSED INFORMATION ON NYSTAGMUS?	NUMBER OF RESPONDENTS (*N* = 111)	PERCENTAGE (%)

Nystagmus Network	69	62%

Online search engine	66	59%

Hospital eye clinic	49	44%

Social media including Twitter and Facebook	28	25%

Sight loss charities	20	18%

Specialist teachers for visually impaired pupils	17	15%

High street or hospital optometrist	13	12%

ECLO	12	11%

British and Irish Orthoptic Society	10	9%

Other professional bodies	6	5%

Researched medical literature	5	5%

Other clinicians (ENT team or neurologist)	4	4%

Royal College of Ophthalmologists	3	3%

Specialist nystagmus unit (e.g., Leicester)	3	3%

Other charities	2	2%

Work colleague	1	1%

Job	1	1%

**Total**	**309**	


All 111 respondents completed question 9. Respondents could select multiple options. A total of 309 responses were recorded.

Most respondents reported there were no barriers to accessing the Nystagmus Information Pack (94.5%; question 10). The small number that reported there were barriers (five respondents) cited poor vision (*n* = 3) or lack of internet access combined with poor vision (*n* = 2) as the barriers that existed for them. One clinician responded ‘unsure’ due to experiencing blocked internet access to the Nystagmus Information Pack at their hospital site (Table 6).

**Table 6:** Barriers to accessing the Nystagmus Information Pack.


QUESTION 10: ARE THERE ANY BARRIERS TO YOU OR OTHERS ACCESSING THE NYSTAGMUS INFORMATION PACK?	NUMBER (*N* = 110)	PERCENTAGE (%)

No	104	94.5%

Yes	5	4.5%

Unsure	1	1%

**Total**	**110**	**100%**


The majority of respondents who had not accessed the Nystagmus Information Pack (45%) reported that additional information about nystagmus would be useful to include in an information pack about nystagmus (question 11; Table 7). Including information on treatment options, living with nystagmus during the different stages of life, employment and educational implications, and a child-friendly section were suggested additions to the nystagmus information.

**Table 7:** Additional information to be included in an information pack about nystagmus.


QUESTION 11: DO YOU THINK ANY ADDITIONAL INFORMATION ABOUT NYSTAGMUS IS REQUIRED IN AN INFORMATION PACK ABOUT THE CONDITION?	NUMBER (*N* = 110)	PERCENTAGE (%)

Yes	28	25%

No	45	41%

Don’t know	37	34%

**Total**	**110**	**100%**


### Additional open comments at the end of the questionnaire

All respondents were given the option of adding additional comments at the end of the questionnaire (question 32). Suggestions were made relating to children with nystagmus, the content of the Nystagmus Information Pack, additional versions and accessibility options, and general awareness of the resource. These comments are presented in Table 8.

**Table 8:** Additional open comments and suggestions (question 32).


THEME	COMMENTS MADE BY PARTICIPANTS

Children and school	Developing a child friendly version *

	Developing a video resource to show to schoolchildren *

	Including information about what is helpful for children with nystagmus in school

	Including information for teachers

Content	Would like examples of people living with nystagmus

	Including information on emotional and mental health

	Including information that not all nystagmus is the same and highlighting that some people with nystagmus can have good vision

	Including information about nystagmus specialists in the UK and optometrists specialising in nystagmus

	Including information on Meniere’s disease and links to Meniere’s groups

	Comments made about specific wording used in some parts relating to nystagmus frequency and the actions of mydriatic drugs

Additional versions	Having a shortened leaflet version that could be easily given to patients

	Having a complete version available for download, rather than just individual sections

	Additional comments relating to children shown above *

Accessibility	Having accessibility options when viewing the Nystagmus Information Pack online, including different background colours

	Having a large-print version

Awareness	Need to increase awareness of the Nystagmus Information Pack as a resource

	Suggestion for international groups to share resources and information


## Discussion

The Nystagmus Information Pack (2017) was developed to improve patient, family, and clinician access to high-quality information about nystagmus, which had been highlighted as important following a questionnaire aimed at orthoptists (Bjerre et al. 2018). This evaluation of the Nystagmus Information Pack has highlighted that further improvements in the content and the accessibility of this information should be considered.

The questionnaire respondents in this study were more diverse than the original questionnaire that only gathered information about the accessibility of written information about nystagmus from orthoptists (Bjerre et al. 2018). The respondents included a wide variety of clinicians, people with nystagmus, family members, teachers, and other professionals (Table 1). The results of this questionnaire are therefore more representative of people and professionals who are aware of nystagmus, rather than just orthoptists. However, it is acknowledged that we do not have an equal number of responses from the different groups to which the questionnaire is aimed at. Whilst the respondents were primarily from the UK, several responses were from outside the UK. This highlights that people may seek information about nystagmus from a range of international sources.

### Content

Participants reported that the content of the Nystagmus Information Pack was mainly good. Some areas for improvement and several specific suggestions on how the content could be improved were made (Table 8). It is important to highlight that whilst respondents gave their opinions on the content of the Nystagmus Information Pack, we did not evaluate their understanding of the content or how they applied the information to their own situation or circumstances. Both considerations have been highlighted as important factors when considering health information from the patient’s perspective (Dey 2004).

Several respondents suggested including experiences of others living with the condition and information about emotional and mental health. This finding highlights the importance of considering the different ways nystagmus can affect a person and not just their vision. This has also been reported by McLean, Windridge & Gottlob (2012) in their qualitative study exploring patient perceptions of living with nystagmus. Physical and social restrictions of movement, standing out and not fitting in, negative feelings about one’s inner self, and negativity about the future and relationships were all reported to occur in nystagmus, in addition to poor vision. A quality of life questionnaire has been developed for nystagmus (McLean et al. 2016); however, to our knowledge, it has not yet been used to evaluate the impact of providing information about nystagmus or interventions for nystagmus.

### Accessibility

Only 30.6% of respondents had accessed the Nystagmus Information Pack, with most respondents being unaware it existed. This highlighted that greater promotion and advertising of the resource were warranted, even amongst people and professionals who were aware of nystagmus. Whilst it can be tempting to assume more information about a condition is needed, existing resources could be promoted or shared more widely. Signposting patients to reliable sources of information and improved communication between health and social services and the charitable sector have both been suggested as important to ensure patients can access reliable health information (Colledge et al. 2008). Sharing resources and information about nystagmus across international groups was also suggested by a respondent (Table 8) as an additional comment.

Encouragingly those that had not accessed the Nystagmus Information Pack reported they had received information about nystagmus from a wide range of sources and often multiple different sources (Table 5). This questionnaire did not investigate when people had received information about nystagmus. Additionally, the Nystagmus Information Pack has only been available since 2017. It is therefore not possible to report whether the Nystagmus Information Pack has improved accessibility to information or has been a more commonly used source of information in recent years.

Most respondents reported they did not experience barriers in accessing the Nystagmus Information Pack; however, poor vision was a barrier for a small number (Table 6). Presenting information visually can result in poor access to information for those with vision impairment, leading to frustrations and a loss of independence (Kaye & Marriot 2010). A report from RNIB Scotland (Thurston & Thurston 2010) highlighted the poor accessibility of health information for those with visual impairment. Only 10% of communications (from all health services) were delivered in the preferred reading format for sight-impaired or severely sight-impaired individuals. This resulted in difficulties in accessing and experiencing health care and taking prescribed medications and a heavy reliance on others to enable access to health information (Thurston & Thurston 2010). Whilst this questionnaire did not specifically ask whether respondents had a preferred format for receiving information, it is acknowledged that the Nystagmus Information Pack is currently a written resource, using 14-point font (Verdana), presented as a PDF on a web page. This format may not be accessible to all with nystagmus or vision impairment. Having a large-print version or options to adjust the background (Table 8) were both suggested as alternative formats. People with nystagmus reported a preference for website-based information, videos, and PDF (Table 3). In the future, a range of different formats should be considered when updating the Nystagmus Information Pack.

### Other sources of nystagmus information

In 2018, Bjerre et al. reported 33% of orthoptists surveyed did not have nystagmus information to give to patients. This scenario may have changed following the introduction of the Nystagmus Information Pack. However, respondents to this questionnaire (who had not accessed the Nystagmus Information Pack) reported they had accessed nystagmus information from multiple sources and a wide range of other sources (Table 5). This suggests that despite information not always being given at orthoptic or eye clinic appointments, some patients find nystagmus information using their own searches. This is a view supported by Gummer et al. (2020), who reported 47% of their questionnaire respondents preferred social media and charity websites and social media streams as their preferred sources of nystagmus information.

The respondents to this questionnaire differed in their view on the value of including ‘patient stories’ in the Nystagmus Information Pack (Table 8). Some suggested individual patient accounts as a positive addition, whilst others highlighted that individual experiences and level of vision in nystagmus can be quite varied. Charities such as Nystagmus Network and RNIB often include patient experiences within their information; therefore, consideration needs to be given to avoiding duplication of information and resources. Gummer at al. (2020) reported that awareness of nystagmus support groups and charities was lower than expected, but those receiving support found it useful. This remains an area for clinicians and the authors of information resources to be aware of, as signposting to other sources of nystagmus information and support may be preferable to including additional information on individual experiences in the Nystagmus Information Pack.

### Limitations and directions for further research

Limitations to this study include the small number of respondents who have accessed the Nystagmus Information Pack (*n* = 49) in comparison to those who had not (*n* = 111). This limited our ability to analyse the resource content. Data was not collected on respondent ethnicity, socioeconomic status, or whether the first language was English. This limited our ability to analyse whether these factors affected understanding and interpretation of the Nystagmus Information Pack.

The questionnaire approach also limited the ability to fully or more deeply explore patient experiences using the Nystagmus Information Pack, the reasons for lack of awareness of the resource, and how barriers to accessing it may be overcome. The questionnaire results do not allow conclusions to be drawn about whether the Nystagmus Information Pack has enabled more orthoptists to be able to give patients with nystagmus information about their condition. In the future, research should evaluate not just the content and accessibility of patient information such as the Nystagmus Information Pack but also the understanding of the information by the reader or consumer. The impact of providing patient information should be measured or evaluated, in particular whether the aims of providing the information were actually achieved and how much participants’ prior knowledge of nystagmus influenced their responses. Qualitative research methods could be used to explore patient understanding of both the condition itself and the information provided about nystagmus.

## Conclusion

The Nystagmus Information Pack has been available since 2017. Participants reported the content was mainly good; however, some suggestions were made on information that could be updated or included in the future. The accessibility of the Nystagmus Information Pack was mainly good, for those who were aware of the resource. Consideration needs to be given to the format of the information within the Nystagmus Information Pack to avoid those with poorer vision not being able to access it. Greater promotion of the resource to those with nystagmus or with an interest in the condition is required, as a surprising number of respondents were unaware of the resource.

## Additional File

The additional file for this article can be found as follows:

10.22599/bioj.269.s1 Appendix 1. Questions in the online questionnaire.
