# Supplementary material for: Evaluation of the Nystagmus Information Pack
Source: Br Ir Orthopt J. 2022 Sep 15;18(1):111–20. doi: 10.22599/bioj.269 (PMC9479666; doi:10.22599/bioj.269)
Supplement: Appendix 1. — Questions in the online questionnaire. [file bioj-18-1-269-s1.pdf]

Appendix 1. Questions in the online questionnaire

1. Consent question

2. What is your relation to nystagmus? Please select all that apply.
- I have nystagmus
- I am the family member of someone with nystagmus
- I teach people with nystagmus
- I am an orthoptist, optometrist or ophthalmologist
- I am an Eye Clinic Liaison Officer (ECLO)
- I am another health professional
- I work for a sight loss/disability organisation
- Other - please specify:

3. In which area are you based? Please select all that apply.
- England
- Scotland
- Wales
- Northern Ireland
- Republic of Ireland
- Other - please specify:

4. Please describe your region or area more specifically (e.g. South Yorkshire):

5. In which format would you prefer information about nystagmus to be presented? Please select all that apply.
- Video
- Audio
- Website
- PDF
- Print
- Don’t know
- Other - please specify:

6. Why? (optional)

7. Have you previously accessed the Nystagmus Information Pack produced by the University of Sheffield?
- Yes
- No

If answered No - go to question 8. Answer questions 8 - 12.
If answered Yes - go to question 13. Answer questions 13 - 31.

8. If no, why have you not accessed the Nystagmus Information Pack? Please select one of the options.
- Not heard of it
- Have heard of it, but unable to find it

- Have heard or it, but did not need to access it
- Have heard of it, but it is not accessible to me
- Other - please specify:
- Additional information (optional):

9. Where have you previously accessed information on nystagmus, if at all? Please select all that apply.

- I haven't previously accessed information on nystagmus
- Search engine, such as Google or Yahoo
- Social media, such as Facebook or Twitter
- YouTube
- Other website - please specify:
- Hospital eye clinic
- High street optometrist (optician)
- Eye Clinic Liaison Officer/Sight Loss Advisor
- Specialist teachers for visually impaired pupils
- Nystagmus Network
- National sight loss charity, such as RNIB, Victa or Look - please specify:
- Local sight loss charity - please specify:
- Other charity or support group - please specify:
- British and Irish Orthoptic Society
- Royal College of Ophthalmologists
- Other professional body - please specify:
- Overseas national sight loss charity - please specify:
- Overseas local sight loss charity- please specify:
- Other - please specify:

10. The Nystagmus Information Pack is available for free on the University of Sheffield website. Are there any barriers to you or others accessing the Nystagmus Information Pack?

- No
- Yes - internet access
- Yes - language barrier
- Yes - vision
- Other - please specify:

11. The Nystagmus Information Pack contains seven different sections, shown below.
Part 1 - an introduction to nystagmus
Part 2 - infantile nystagmus
Part 3 - acquired nystagmus
Part 4 - what to expect at eye clinic appointments
Part 5 - treatment options and long-term outcomes
Part 6 - useful contacts and information
Part 7 - information about nystagmus for families

Do you think any additional information about nystagmus is required in an information pack about the condition? Please select one option:

- Yes
- No

- Don’t know

12. Why? (optional): (free text)

Continue to question 32.
……………………………………………………………………………………………………...

If answered Yes to question 13.

13. If yes, where did you hear about the Nystagmus Information Pack? Please select all that apply.

- Search engine, such as Google or Yahoo
- Social media, such as Facebook or Twitter
- Hospital eye clinic
- High street optometrist (optician)
- Eye Clinic Liaison Officer/Sight Loss Advisor
- Specialist teachers for visually impaired pupils
- Nystagmus Network
- National sight loss charity, such as RNIB, Victa or Look - please specify:
- Local sight loss charity - please specify:
- Other charity or support group - please specify:
- British and Irish Orthoptic Society
- Royal College of Ophthalmologists
- Other professional body - please specify:
- Overseas national sight loss charity - please specify:
- Overseas local sight loss charity- please specify:
- Other - please specify:

14. If you wanted to access it again, would you know how to find it? Please select one option.

- Yes
- No
- Don’t know

15. Additional information (optional): (free text)

16. Where else have you previously accessed other information on nystagmus, if at all? Please select all that apply.

- I haven’t previously accessed information on nystagmus
- Search engine, such as Google or Yahoo
- Social media, such as Facebook or Twitter
- YouTube
- Other website - please specify:
- Hospital eye clinic
- High street optometrist (optician)
- Eye Clinic Liaison Officer/Sight Loss Advisor
- Specialist teachers for visually impaired pupils
- Nystagmus Network

- National sight loss charity, such as RNIB, Victa or Look - please specify:
- Local sight loss charity - please specify:
- Other charity or support group - please specify:
- British and Irish Orthoptic Society
- Royal College of Ophthalmologists
- Other professional body - please specify:
- Overseas national sight loss charity - please specify:
- Overseas local sight loss charity- please specify:
- Other - please specify:

17. The Nystagmus Information Pack is split into seven parts, with each part addressing a separate topic related to nystagmus. Which parts have you accessed? Please select all that apply.
    - Part 1 - An introduction to nystagmus
    - Part 2 - Infantile nystagmus
    - Part 3 - Acquired nystagmus
    - Part 4 - What to expect at eye clinic appointments
    - Part 5 - Treatment options and long-term outcomes
    - Part 6 - Useful contacts and information
    - Part 7 - Information about nystagmus for families
    - Don’t know

18. Do you find the organisation of the Nystagmus Information Pack into separate parts helpful? Please select one option.
    - Yes
    - No
    - Don’t know

19. Why? (optional): (free text)

20. From your experience of using the Nystagmus Information Pack, do you think the language it uses is easy-to-understand and can be accessed by people without a medical background? Please select one option.
    - Yes
    - Sometimes
    - No
    - Don’t know

21. Why? (optional): (free text)

22. Thinking about the content of the Nystagmus Information Pack. What do you think about the information in the pack? Please select all that apply.
    - It contains information that is not needed
    - It contains information that is misleading, inaccurate or ambiguous
    - Important information is missing
    - All the information is needed
    - All the information is appropriate

23. Why? (optional): (free text)

24. Thinking about the content of the Nystagmus Information Pack. What do you think about the length of the pack? Please select one option.

- It is too long
- It is too short
- The length is appropriate

25. Why? (optional): (free text)

26. Thinking about the content of the Nystagmus Information Pack. What do you think about the detail of the pack? Please select one option.
    - It is too detailed
    - It is not detailed enough
    - The level of detail is appropriate

27. Why? (optional): (free text)

28. Thinking about the content of the Nystagmus Information Pack. What do you think about the diagrams and illustrations in the pack? Please select one option.
    - The diagrams and illustrations are helpful
    - The diagrams and illustrations are not helpful
    - The diagrams and illustrations are appropriate

29. Why? (optional): (free text)

30. Do you think it would be of benefit to include case studies and examples of other peoples' experiences in the Nystagmus Information Pack?
    - Yes
    - No
    - Don't know

31. Why? (optional): (free text)

Continue to question 32.
………...

32. Finally, please add any further comments about the Nystagmus Information Pack you would like to share with the researchers below:
(free text box)

END OF QUESTIONNAIRE
